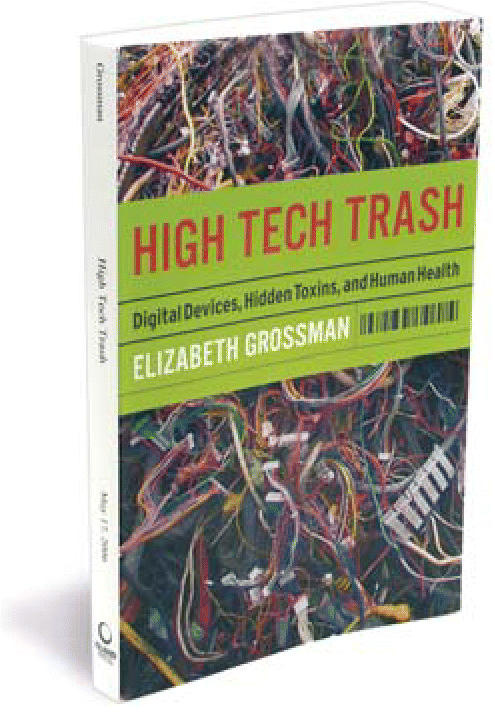# High Tech Trash: Digital Devices, Hidden Toxins, and Human Health

**Published:** 2006-08

**Authors:** Andreas Sjödin

**Affiliations:** Andreas Sjödin is the laboratory chief of the Combustion Products and Persistent Pollutants Biomonitoring Laboratory at the National Center for Environmental Heath at the Centers for Disease Control and Prevention. This laboratory conducts measurements for brominated flame retardants and other persistent organohalogens and polycyclic aromatic hydrocarbon metabolites in human biological specimens for epidemiologic studies such as the National Health and Nutrition Examination survey

By Elizabeth Grossman

Washington, DC:Island Press, 2006. 334 pp. ISBN: 1-55963-554-1, $25.95

In *High Tech Trash*, Elizabeth Grossman traces the effects of the high-tech computer industry on the environment, from the raw materials to the chemicals and solvents used to produce silicone chips as well as other persistent organic compounds used to produce high-tech equipment. Grossman then describes the subsequent recycling of electronics for reuse as well as the recovery and recycling of raw materials such as copper, zinc, gold, and plastics.

This journey through electronics, which formerly had been recognized as a “clean” industry, begins in the mines that supply many of the raw materials—including copper, aluminum, lead, gold, zinc, nickel, tin, silver, and iron—used in modern electronics. Grossman then discusses the different chemicals and solvents used in the production of silicone chips. She gives a close-up view of the electronics industry, recognizing the improvements in chemical safety from the early days when many of the tasks involving solvents such as trichloroethylene (TCE) and trichloroethane (TCA) were performed manually. These improvements have helped ensure that workers’ exposures to chemicals have been substantially reduced in the automated factories of today. Grossman further points out that the groundwater at current Superfund sites is now contaminated with TCE and TCA as a result of leaks and spills that occurred in the early days of the electronics industry. The fact that several residential areas once drew drinking water from these sites and that vapors may pass upward from soil to air has, says Grossman, affected thousands of people.

In her review of the available literature on polybrominated diphenyl ethers (PBDEs), Grossman discusses the likely link between human exposure to PBDEs and the high levels of PBDEs in residential dust. Unfortunately, Grossman frequently uses the generic term “PBDEs” rather than specifying which congeners are present in the technical-grade preparation of the PBDEs she discusses. Primarily, technical-grade octabromodiphenyl ether (octaBDE) and decabromodiphenyl ether (decaBDE) are used in the plastic housings of electronics, whereas technical-grade pentabromodiphenyl ether (pentaBDE) is used primarily in polyurethane to manufacture foam and padding materials. The congeners present in the pentaBDE mixture have been shown to accumulate in biological tissues, but the PBDEs in octaBDE and decaBDE have much shorter half-lives in people and are unlikely to biomagnify, although they are detectable in many human tissues.

The chapters on recycling and on the flow of electronic waste from consumers to landfills and the export of waste to developing countries are well written and detailed. For example, Grossman states that the United States discards enough electronic waste annually to cover a football field a mile high and that of this waste, only 10% is recycled for materials recovery. Most of the waste goes to landfills or to waste incinerators.

Grossman describes the handling of electronic waste that is exported from developed countries into developing countries and the tremendous environmental impact this waste has had in certain countries. For example, in Taizhou in southern China, circuit boards containing lead, flame retardants, and plasticizers are melted for the recovery of metals in uncovered pans only steps away from the workers’ dormitories. In addition, the author offers an interesting description of the different recycling processes used by industrial companies such as Boliden Mineral AB in Sweden and Noranda in the United States. These companies have discovered a “rich ore stream” in the circuit boards they “mine” for metals.

Clearly, the increasing tide of electronic waste will be brought under control only if all parties involved (manufacturers, legislators, consumers, and recyclers) implement improvements such as reducing the amount of hazardous materials used in the manufacture of electronic products, producing electronics that are easier to disassemble for recycling, enacting legislation governing the electronics and recycling industries, and ensuring that consumers are informed about the issues associated with the production of electronics and with electronic waste so they can make educated decisions about the issues. Unfortunately, we have just now begun to address these issues, and changes will not be quick or easy.

## Figures and Tables

**Figure:**